# Memory reconsolidation: a proposed change mechanism for the arts therapies

**DOI:** 10.3389/fcogn.2025.1518743

**Published:** 2025-05-15

**Authors:** Noah Hass-Cohen, Jennifer C. Clay

**Affiliations:** ^1^California School of Professional Psychology, Alliant International University, Alhambra, CA, United States; ^2^Art Therapy Psychology, Dominican University of California, San Rafael, CA, United States

**Keywords:** arts therapies, brain, neuroaesthetics, ATR-N, memory reconsolidation, common therapeutic factor, relational neuroscience, trauma

## Abstract

It is proposed that memory reconsolidation (MR) processes are a common therapeutic change mechanism for arts therapies and that arts therapies' processes uniquely facilitate the MR of autobiographical and distressing memories. This study aims to review memory reconsolidation, identify the necessary neuroscientific conditions for therapeutic MR, and examine the alignment between Art Therapy Relational Neuroscience (ATR-N) principles, interventions, and MR conditions. A comprehensive description of two ATR-N MR case drawing protocols is provided along with application guidelines based on two decades of research on the ATR-N drawing protocols.

## Introduction

Autobiographical narratives and memories are thought to be created by a dynamic comparison of past to current memories (Waters, 2014). The purpose is to enable salient future decision-making. Neuroplasticity facilitates this comparison and modification of autobiographical memories by the process of memory reconsolidation (MR; Tronson and Taylor, 2007). MR has an evolutionary role; it continuously mediates responses to past and current disruptive memories and future threats (Björkstrand et al., 2017).

MR involves memory recall and then modification. Upon memory recall, proteins in the memory and fear centers of the brain become pliable (Nader et al., 2000). Then over a few hours, the subsequent resynthesis of proteins supports the modification of previously consolidated memories (Hardt et al., 2010). Should this process generalize, newly formed, reconsolidated memories could be retrieved instead of the original memory being recalled (Lane et al., 2015). In psychotherapy, the aim is to cultivate coherent autobiographical narratives by mitigating and facilitating the forgetting of distressing memories (Agren, 2014; Ecker et al., 2024, 2015; Lane et al., 2015; van den Berg et al., 2014).

For the current study purposes, MR has been defined as a positive and novel modification of memory and strengthening of disturbing memories, rather than a negative process that we have labeled rekindling or reconditioning. The term *disturbing memories* has been defined as ranging from mild to severe (Martalek et al., 2024), yet distinctions are made to differentiate traumatic fragmented memories from non-traumatic autobiographical memories.

Neurobiologically, traumatic autobiographical memories have been categorized as relived memories, meaning immersive, intrusive, and non-voluntary vs. voluntarily relived and self-initiated (Brewin, 2018; Kearney and Lanius, 2024; Lanius and Kearney, 2024). It is crucial to note that seemingly non-traumatic or mildly stressful autobiographical memories may unknowingly include elements of traumatic and fragmented experiences (Barreiro et al., 2013; Hass-Cohen and Clyde-Findlay, 2009).

Psychologically, recall triggers the emotions associated with the original memory. If these emotions are distressing, the reactions may be modified by safety-oriented restorative affective and cognitive experiences that support forgetting, new learning, and resilient coping (Lane and Nadel, 2020). Over time, mental health outcomes might be improved by such interventions that disrupt unpleasant, stressful, or fear-based traumatic memories and then facilitate memory updating with non-threatening information (Burback et al., 2024). Applied MR processes may result in long-lasting changes to habitual responses to distressing and traumatic memories (Schiller et al., 2010; Schiller, 2022).

The MR-based Art Therapy Relational Neuroscience (ATR-N) guidelines have been supported by research and include (a) safely identifying which experiences are maintaining the issues or symptoms; (b) juxtaposing these experiences with novel evidence that can generate experiential disconfirmation, that is, predictive errors, and symptom transformation by means of a corrective resilient emotional reaction; and (c) promoting potential permanent updating of the memory (Ecker et al., 2024; Hass-Cohen, 2024; Hass-Cohen et al., 2021, 2022a,b; Levy and Schiller, 2021; Vaisvaser, 2021). These have guided the creation of the ATR-N protocols, which are described and summarized in this article.

## ATR-N and MR

Research on ATR-N-based MR has examined specific theoretically developed four- and three-drawing protocols (Hass-Cohen and Clyde Findlay, 2019a,b; Hass-Cohen, 2016, 2018, 2024) quantitatively (Hass-Cohen et al., 2014; Hass-Cohen, 2018; Hass-Cohen et al., 2021) and qualitatively (Hass-Cohen et al., 2022c,b). While not identified as MR, an investigation of memory-based pictorial outcomes has been suggested for arts therapies (Gerge and Pedersen, 2017). Other MR-relevant art therapies outcomes such as reduced arousal and increased emotional positivity and self-efficacy (Spiegel et al., 2006), as well as resilience (Naff, 2014), were reported. Associated therapeutic factors have been identified as symbolic expression, containment, and artistic pleasure (Smith, 2016); sensory-tangible artwork characteristics (Hass-Cohen and Loya, 2008); and imagination and creativity (Lahad et al., 2010).

**Figure 1:**
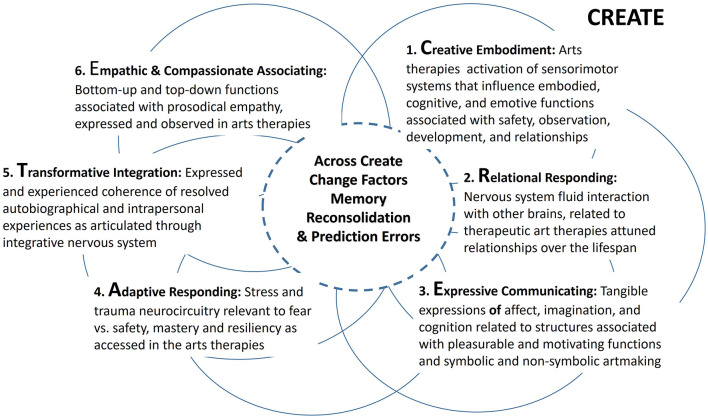
Venn diagram of the six overlapping principles/factors of Art Therapy Relational Neuroscience with partial descriptions. CREATE: (1) creative embodiment in action, (2) relational responding, (3) expressive communicating, (4) adaptive responding, (5) transformative integration, and (6) empathizing and compassion. Partial descriptions from Hass-Cohen and Clyde Findlay (2015).

Hass-Cohen (2024) has aligned MR constructs and outcomes with the six ATR-N CREATE principles, which are referenced throughout this study. The CREATE principles are (a) creative embodiment in action, (b) relational responding, (c) expressive communicating, (d) adaptive responding, (e) transformative integration, and (f) empathizing and compassion (Figure 1).

The ATR-N CREATE principles evolved from the transliteration of neuroscientific research and the examination of art therapies clinical practices (Hass-Cohen and Loya, 2008; Hass-Cohen and Clyde Findlay, 2015; Hass-Cohen, 2024). The principles are ATR-N clinical and research guiding factors. They overlap, as they are interconnected rather than prioritized factors or treatment stages, structural networks, and functions, among others (Hass-Cohen, 2008; Hass-Cohen and Clyde Findlay, 2015). Aesthetic-based neural network–based factors (Vaisvaser et al., 2024), as well as body–mind factors (Czamanski-Cohen and Weihs, 2016), have been identified as mechanisms of change in arts therapies. However, the present claim differs by claiming that MR dynamics are the core and common ATR-N change factor for any ATR-N approaches. In support of these theoretical claims, the relevant literature on MR and the conditions for MR are reviewed, and two research-supported ATR-N drawing protocols of a case are illustrated. Through these case studies' descriptions, the intent is to illustrate a potential application of the ATR-MR protocol and highlight ATR-N-based grounded theory concepts. The case illustrations are not intended to demonstrate clinical efficacy. Rather, the purpose is to provide a summative description and discussion of over a decade of research on ATR-N drawing protocols and conditions (Hass-Cohen, 2024). The proposal is that ATR-N-MR practices support implicit and explicit recontextualization and modifications of recalled memories (Ghilardi et al., 2009; Jacques and Schacter, 2013; Hass-Cohen, 2018; Hass-Cohen et al., 2021, 2022a,b; Lenormand et al., 2024).

Moreover, it is likely that expressive arts interventions assist in mediating risks associated with the recall of distressing memory. One way in which ATR-N practices support safety is by (a) increasing cognitive therapeutic awareness of vulnerability associated with the reconditioning and rekindling of traumatic distressing memories and (b) providing resources and options to master this vulnerability. The general hypothesis is that engrossing and rewarding creative arts experiences recruit the synchronization of the limbic subcortical system, the autonomic nervous system, and then central nervous system functions (Christensen and Gomila, 2018; Yamasaki and Takeuchi, 2017). When this synchronization is ordered and coherent, it likely supports MR (Ecker et al., 2024; Hass-Cohen, 2016, 2018, 2024).

## MR

**Figure 2:**
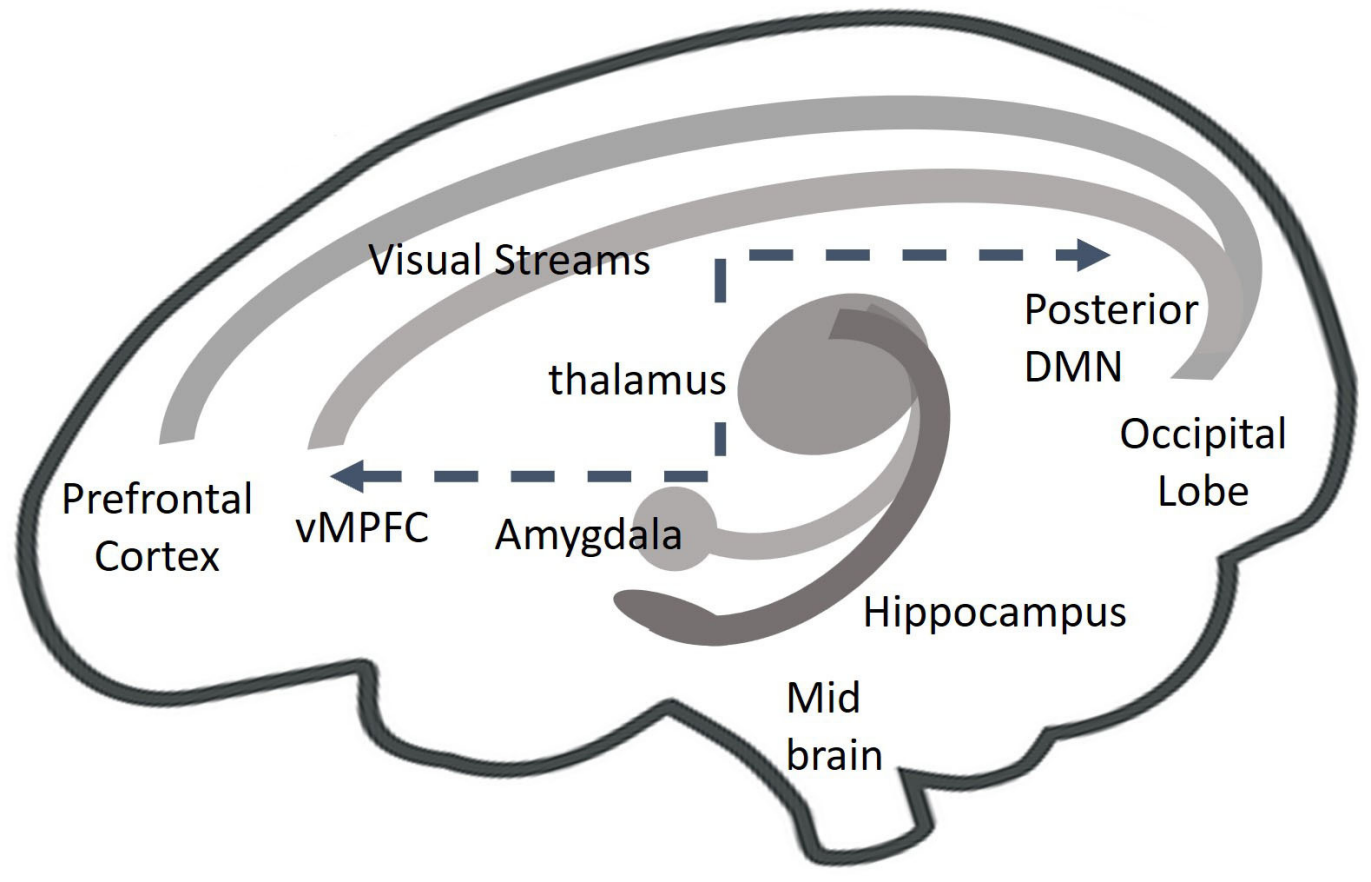
Autobiographical ATR-N memory processing of episodic memories, such as fear memories, activates a dynamic interplay of amygdala and hippocampus functions, dorsal and ventral visual processing stream. This information coalesces in the prefrontal cortex. specifically in the dorsal lateral prefrontal cortex. Thalamic processing of sensory information further regulates the interconnectivity of cortical and subcortical areas. When traumatic memories are recalled, hyper connectivity occurs between the posterior area of the default mode network (DMN) and the sensory- motor system, including and not limited to, subcortical areas such as the including and not limited to, subcortical areas such as the mid brain.

Throughout a person's lifespan, personal memories constantly change and contribute to the autobiographical sense of self in the past, present, and future (Moscovitch and Gilboa, 2022; Tulving and Markowitsch, 1998). Specifically, changes in episodic and semantic memories, which make up autobiographical memories, consolidate into short- and long-term memories and reconsolidate with ongoing recall (Nader et al., 2000). Such processes have been linked to the brain's neuroplasticity, that is, the potentiation of strengthened and alternative neuropathways and the rebirth of new neurons in the memory center of the brain (Dieni et al., 2019; Jasey and Ward, 2019; Kempermann et al., 2018). This dynamic autobiographical processing activates a network of neuropathways. A psychologically relevant list includes the amygdalae (fear) and hippocampal (memory) formations (Kandel et al., 2014), visual system pathways (Brewin, 2018), the default mode network, thalamic networks (Venkataraman and Dias, 2023), and multiple subcortical and cortical sensory systems interfaces (Kearney and Lanius, 2024; Figure 2).

## Memory and neuroplasticity

**Figure 3:**
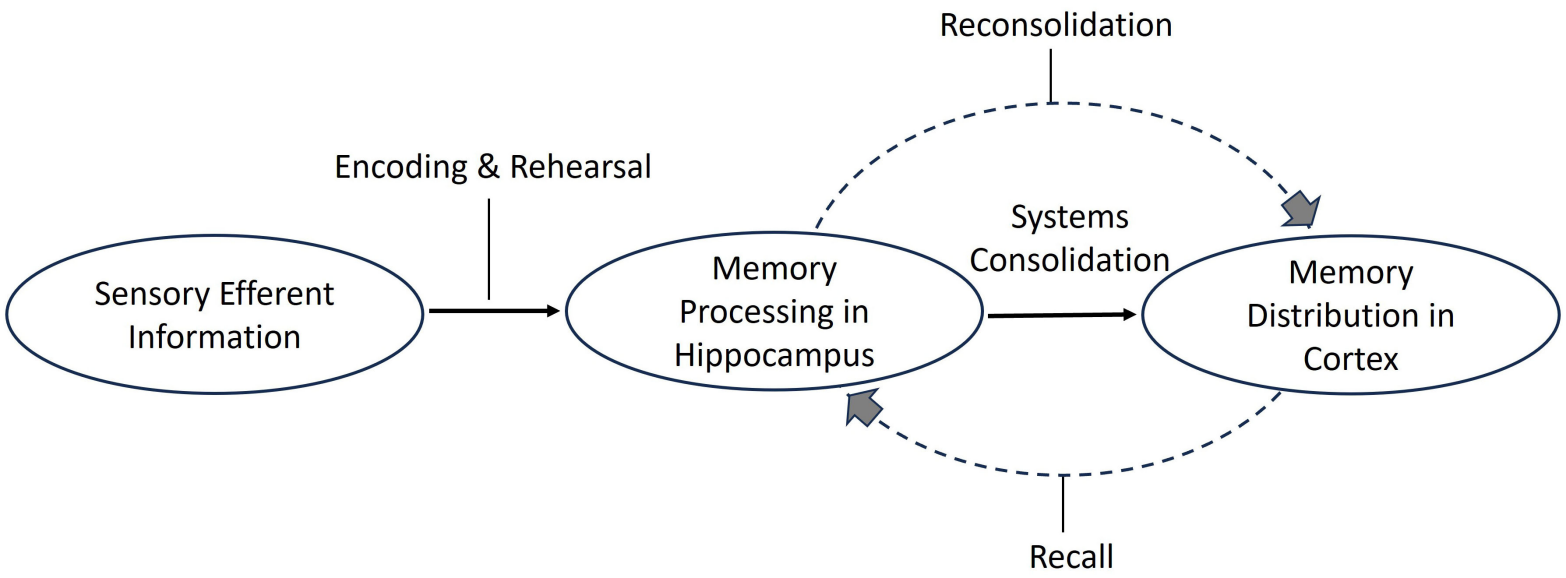
Memory consolidation and reconsolidation. Schematic illustration of memory consolidation and reconsolidation. Memory reconsolidation involves modifying consolidated memories (adapted from multiple resources).

Memories are held in the hippocampal–amygdala formation for approximately 2 years before they are distributed cortically and embedded in long-term memory (Kandel et al., 2014; Schwabe et al., 2014). In contrast, reconsolidation occurs a few hours after existing when consolidated memories are recalled and recruited back to hippocampal areas (Hardt et al., 2010), thus presenting an opportunity for existing memories to be updated (Elsey et al., 2018). Following recall and modification, reconsolidation occurs within a 4- to 6-h window in the hippocampus and amygdala (Nader et al., 2000). Through the synaptic potentiation and re-synthesization processes that occur within this time frame, modification of the original memory trace strengthens and reorganizes future retrieval pathways (Aimone et al., 2014; Bellfy and Kwapis, 2020). The finding that protein changes were chemically different for initial systems consolidation compared to memory recall, and reconsolidation has supported these neuroplasticity claims (Tronson and Taylor, 2007; Figure 3).

This neuroplasticity likely occurs in the hippocampus by (a) repeated exposure and engagement with novel yet salient inputs usually associated with predictive errors, meaning a contrast between previous experiences and current ones and priming older information with novel aspects via protein changes, and (b) the inhibition of repeated recall of negative narratives and exposure to contextual cues that facilitate other modifications (Schwabe et al., 2014). During the MR time frame, the modified memory must be protected from such negative, that is, retroactive interferences (Sekeres et al., 2024). Retroactive inferences, often associated with negative bias and habits or interpersonal negative feedback from others, occur after modification. In contrast, proactive interferences, again habitual negativity, constrain the potential for modification upon initial retrieval and recall. Both retroactive and proactive inferences act to confirm the original consolidated or conditioned memory of the experience.

Interventions have furthered differential understanding of MR mechanisms. For example, episodic personal memories are likely impacted separately from their semantic-related information (Kindt et al., 2009; Kindt and Elsey, 2023). While amygdala-driven fear responses were modulated by administering medication (Bitencourt and Takahashi, 2018; Lonergan et al., 2013), neutral and factual memories of the same event were not changed within the hippocampal formation (Schwabe et al., 2014). These findings supported ethical foundations for MR psychotherapy interventions, which focus on changing fear or negative responses to facts.

Additional characteristics and contextual dimensions of different types of memory may impact memory modification. Long-term memories are likely more resistant to change as they are stable and vague, whereas more recent vivid memories may be more susceptible to modification (Wang, 2018; Brewin, 2018). Research on memory decay and interference, as well as on cognitive capacity, proposed that (a) less-used memories can fade, that is, soften, over time and (b) new memories can then possibly interfere with the salience of old memories (Constant and Kerzel, 2025; Malleret et al., 2024). Others have suggested that other factors may be impacting these issues, such as whether the memories are implicit or explicit, their degree of vividness, the level of arousal during recall, and whether they are semantic-factual or episodic-personal (Kim et al., 2021). For example, if the threat is not impending, it may be more easily modified than an imminent threat (Levy and Schiller, 2021). Memories in the hippocampus are encoded and consolidated according to contextual temporal dimensions, such as the past, the present, or the future (Montagrin et al., 2024). The hippocampal formation also contextualizes personal events with spatial-visual characteristics (Eichenbaum, 2017; Smith and Bulkin, 2014; Wang, 2018). These hippocampal functions have been associated with memory formation, such as remembering and forgetting (Aimone et al., 2014), and are likely advantageous to MR-supported clinical work (Small, 2021).

This is pertinent for ATR-N MR-tangible practices that invite both engaging with and looking at spatial, sensory, and playful environments. The assumptions are that MR is facilitated by characteristics of ATR-N practices that provide access to implicit and older memories and tame the potential rekindling of conditioned threats. These characteristics likely aid in memory recall and modification regardless of memory age and type, due to (a) the vivid characteristics of the artwork, (b) threat mediation related to the symbolic nature of expression (c) containing and pleasurable nature of arts-based work, (d) self-regulating and satisfying experiences related to mastering artmaking, and the expression of implicit memories (e) potential for tangible, visual-spatial organization of autobiographical memories (Hass-Cohen et al., 2018; Lukaschewski et al., 2023).

## Meaning-making and memories

To trigger MR, the recalled memory must be disrupted by a new meaning that contradicts habitual and negative predictive schemas (affective and cognitive) associated with it, or else the recalled experience may be reconditioned (i.e., rekindled; Lane et al., 2015). To do so, an interface between the recalled memory and novel information or meaning is needed (Wichert et al., 2013). Ideally, this interface triggers a prediction error, meaning a different perspective on the old problem or a mismatch with habitual beliefs, understanding, knowledge, experiences, and the like (Lee et al., 2017; Vaisvaser, 2021). Another option, which generates prediction errors, is to interrupt an expected narrative ending (Sinclair et al., 2021). It is also likely that prediction error formations are triggered by emotions (Heffner et al., 2021), such as general positivity (Contractor et al., 2018), optimistic predispositions, and specific relevant optimistic predictions (Wang et al., 2021; Zheng and Mei, 2023).

Anticipatory processes start with the reward center activation, which likely transmits a dopaminergic signal to the prefrontal cortex via the hippocampal–amygdala connections (Levy and Schiller, 2021). Prediction errors activate basal forehead acetylcholine release, which triggers unexpected feedback to the hippocampus and, therefore, disrupts its patterns, allowing for memory modification and updates (Sinclair et al., 2021).

Furthermore, large prediction errors may trigger neurogenesis in the hippocampus (Levy and Schiller, 2021). These represent an imbalance of the dopamine system triggered by the interface of bottom-up and top-down inputs, that is, a novel calculation of motivation and pleasure (Millard et al., 2022). There is also evidence to suggest that large prediction errors dampen sensorial inputs that support the previous experience and enhance different perceptual and cognitive appraisals (Richter et al., 2024). From a psychological perspective, the question is whether the person will accept a large prediction error, meaning a new meaning, when the gap between their previous and current experiences is very large.

Clinically affective and cognitive therapeutic activation of predictive errors and personal reactions to predictive error activations need to be considered. ATR-N interventions and artmaking are uniquely poised to trigger such prediction errors and mediate their acceptance (Hass-Cohen, 2024). The following are some of the possible reasons: (a) Novelty is easily facilitated by pairing a psychosocial issue with the artmaking materials and experiences; (b) artmaking in response to a psychological issue interrupts the expected endings and outcomes of the personal existing narrative; (c) new or contradictory meaning-making is supported by tangible sensory experiences, not only verbally; and (d) therapeutic artmaking and creativity are pleasurable and positive experiences. In summary, it is likely that MR is facilitated through sensorial and tangibly expressed ATR-N creative embodiment practices (Hass-Cohen, 2024).

## Recall arousal and fear

A strong arousal of fear during recall will likely cause re-traumatization by strengthening and conditioning fear-based memories (Brewin, 2018) and hindering the likelihood of memory modification and MR (Hartley et al., 2014). For people with posttraumatic stress disorder (PTSD), a fear reaction to recall is likely relived, meaning experienced as happening right now, which may also contribute to a continuum of responses from a sense of loss of control and hypervigilance (Fitzgerald et al., 2018) to dissociation (King-West and Hass-Cohen, 2008). There can be a vivid sense of no ability to escape from a threat in the past in the present and in the foreseeable future that likely reconditions a feeling of imminent and chronic threat (Sapolsky, 2004; Sege et al., 2023). In this case, survival functions of lower level brain functions (the reptilian system) and the polyvagal dorsal pathway likely activate immobilization, prioritizing physical safety rather than relational connectiveness (Porges, 2021) or new meaning-making. Characteristics of enduring, stable, and reiterative fear response are strongly linked to functions of the hippocampus recall of the salient facts, the activation of the amygdala, and a cascade of catecholamines and hormones (Brewin, 2018). Fear is associated with the activation of the right amygdala (Sergerie et al., 2008).

Specifically, lateral parts of the amygdala associated with avoidance and negativity are triggered and block connectivity to the coping areas, that is, the central and basal areas of the amygdala (Ledoux, 2000; Soeter and Kindt, 2013). This reaction becomes chronically conditioned and has been associated with traumatization, as each time fear is activated by memory, there is a kindling of amygdala lateral pathways (Nader et al., 2000). When a release of catecholamines kindles an amygdala fear response, it also dampens the medial prefrontal cortex (mPFC), the self-center of the brain's cognitive and executive functions (Brewin, 2018). The mPFC is responsible for curbing fear responses (Hayes et al., 2012) and higher level cortical executive functions (Hinojosa et al., 2024). In this case, the generation of prediction errors, critical to MR modification, is constrained due to their partial reliance on cognitive functions (Pedreira et al., 2004; Sinclair et al., 2021).

MR processes may be constrained by intense or severe and chronic fear-based memories yet facilitated by pleasurable memories or those of coping and resiliency (Beckers and Kindt, 2017). Notable is that the same network is activated for both fear and pleasure and seems to be likely associated with the saliency and intensity of the stimulus (Paquelet et al., 2022). When compared to right amygdala function, the interface of left amygdala activation and catecholamines has been linked to coping (Sergerie et al., 2008). Then, in contrast to the recall of inescapable fear, a recall of past escapes or the imagination of a potential escape activates psychological coping. This will then likely activate coping areas of the amygdala and circumvent lateral connections (Hartley et al., 2014). In the same vein, fear-based memories in the amygdala and impaired responses in the ventral mPFC can be modulated by the release of serotonin, the dorsal raphe nucleus of the reward center (Lee et al., 2017). Finally, a meta-analysis review has suggested that a dose of oxytocin, which is released by the pituitary gland in response to social bonding likely has a positive impact on the evaluation and response to threats (Leppanen et al., 2018; Triana-Del Río et al., 2018). So social support, threat control, and positive feelings support coping.

Understanding the neural network activation associated with the MR recall and fear response may shed light on the advantages of engaging in ATR-N art practices, specifically those associated with relational resonance, expressive communication, and adaptive responding principles (Hass-Cohen and Clyde Findlay, 2015; Figure 1).

According to a neuroimaging study, artmaking triggers positive emotions and the reward circuitry (Kaimal et al., 2017). This supports the potential benefits offered by ATR-N MR practices for managing high arousal. Arts therapies work has been associated with tangible pleasure (Czamanski-Cohen and Weihs, 2016; Malhotra et al., 2024), as well as with a symbolic and concrete sense of mastery and control (Hass-Cohen and Clyde Findlay, 2015). Applying practices associated with the adaptive responding principle (Figure 1) may be implicitly or symbolically experienced as an escape route or refuge, potentially stimulating the amygdala's coping-response basal areas, as well as the left amygdala's response (Curl, 2008; Czamanski-Cohen and Weihs, 2016). Creative expression also stimulates the release of serotonin and dopamine (Mayseless et al., 2013; Zaidel, 2014), suggesting the potential advantage of ATR-N practices for modifying fear responses with positive emotions. The therapeutic relationship practiced through principles of relational resonance likely enhances these MR therapeutic factors as relational resonance may optimize the fronto-limbic connectivity associated with positive attachment and the therapeutic relationship (Hass-Cohen and Clyde Findlay, 2015).

## Stress response

The impact of stress responses on MR encompasses multifaceted factors (Sapolsky, 2004; Schwabe et al., 2014). Idiosyncratic contextual factors include age, gender, and psychobiological mental and medical vulnerabilities (James et al., 2023). The magnitude of the stress response to recalled autobiographical memories and relived fragmented PTSD episodic memories impacts cognition and, by extension, MR modification (James et al., 2023; Meir Drexler and Wolf, 2018).

Chronic or severe cortisol release responses impair the hippocampus's memory functions and solidify fearful circuitry; in comparison, milder stressors may help modify the memory by calling attention to its details (Bos et al., 2014; Meir Drexler and Wolf, 2018; Zohar et al., 2011). A dual model of the pathogenic interface between PTSD and stress responses has suggested that cortisol release contributes to a dynamic vicious cycle, where the synaptic loss in the hippocampal and prefrontal areas is accompanied by synaptic gain in the amygdala (Abdallah et al., 2019). This evidence highlights the predominance of the fear response described earlier. The timing of the stress response, whether before or after recall, is also critical for evoking forgetfulness and avoidance or fear of emotional stimuli and requires further investigation (Shields et al., 2017). Prediction error signals critical for cognitive learning associated with MR modification seem to be constrained during acute-stress cortisol and norepinephrine responses to traumatic stress (Carvalheiro et al., 2021; Gerlicher et al., 2018).

The ATR-N adaptive responding interventions differentiate between targeting short- and long-term stress responses and traumatic responses (Hass-Cohen and Clyde Findlay, 2015). These ATR-N practices may mediate these PTSD- and stress-related limitations due to (a) the stress-regulating impacts of perceived internal and external control and mastery induced by the artmaking and (b) the allostatic balancing of reward and stress circuitry associated with the pleasure of creating (Hass-Cohen, 2016, 2024).

## Fragmentation and rumination

As stated, neuroimaging has found support for previous assertations (Brewin, 2018) that traumatic memories for people with PTSD are likely relieved and not remembered (Lanius and Kearney, 2024; Kearney and Lanius, 2024). When compared to people with no PTSD, for people with PTSD, specific brain activations and alterations are likely. Notable was hyperconnectivity between the (a) sensorimotor nervous system, which coordinates and integrates sensory and motor inputs and the midbrain, and (b) the posterior default mode network, associated with arousal, rumination, episodic memory recall, and creativity (Lanius and Kearney, 2024; Kearney and Lanius, 2024; Menon, 2023). Related to these results is that integrative visual, auditory, and motor functions of the midbrain and subcortical management of familiar vs. novel stimuli, contribute to memory formation (Dutt et al., 2021; Szonyi et al., 2019; King and Williams, 2017). Specifically, these brain stem functions associated with the periaqueductal gray triggering act as central modulators of arousal and memories during PTSD (Brandão and Lovick, 2019). Again, catecholamine, specifically dopamine release, may facilitate MR by prioritizing and contextualizing input processing (Shohamy and Wagner, 2008).

Attributes of the sensory and kinesthetic aspects of ATR-N interventions associated with the creative embodiment principle are likely critical to the increased capacity in MR-related memory storage for the following reasons. Art therapy practices involve explicit, expressed gross and fine motor movements, such as touching, bold gesturing, and marking. These co-guide the motor pragmatic execution of the artwork and the expressive affective aspects and heightened excitation of cardiovascular and respiratory organs and structures are enhanced or controlled by cortical frontal and prefrontal areas associated with implicit executive and language functions (Vaisvaser, 2021; Vaisvaser et al., 2024). Furthermore, associated learning triggered by anticipated movement, motion, mirroring, and executive action also likely regulates affective and cognitive function (Cooper et al., 2013; Hass-Cohen and Clyde Findlay, 2015; Heyes and Catmur, 2022; Rizzolatti et al., 2009). This learning is age- and environment-dependent and very malleable to change (Tallman et al., 2022), thus underscoring therapeutic applications for the critical role of attuned art therapy–related neuroscience interventions (Hass-Cohen and Clyde Findlay, 2015).

## Memory storage

Proactive, negative expectations or retroactive reactions such as entrenched negative beliefs or feeling overwhelmed may severely constrain MR modification (Lane et al., 2015). This is due to the temporal neuroplasticity dimensions that persist during a restricted window of 4–6 hours. Both proactive (pre-recall) and retroactive (post-recall) interferences can be implicit, that is, unexpressed or explicitly expressed negative and protective self-biases, which are commonly associated with trauma survivors' self-belief systems (Brown et al., 2019). Within these temporal dimensions, such habitual interferences may circumvent MR.

ATR-N sensory-implicit processing of memories (as in the creative embodiment principle's interventions) may mitigate such implicit and explicit interferences (Hass-Cohen, 2018). ATR-N conversion of non-expressed and expressed memory to tangible arts products creates a new tangible memory. When compared to verbal memory, storage of this multimodal iconic memory, whether visual, tactile, kinesthetic, or auditory, is distributed in multiple networks, thus contributing to an increased capacity for new memories and learning (Fougnie and Marois, 2011). For example, a recall of a traumatic memory followed by a visual intervention (playing a computer game) decreased reports of intrusive memories (James et al., 2015), indicating visual processing advantages (Hass-Cohen and Loya, 2008; Hass-Cohen, 2016, 2024). The hypothesis is that multimodal stored memories will likely (a) be less susceptible to verbal proactive or retroactive interferences, (b) not compete with MR modification, and (c) enhance cognitive functions (Jacques and Schacter, 2013; Luck and Vogel, 2013).

## Coherence

Given the brief review of these MR-related nervous system boundaries, questions arise as to whether there is a nervous system state conducive to MR. It is suggested that the dynamics of this state, between and within cortical and subcortical cerebral fear-based functions (frontal-limbic and reward center processing and the default mode network) and autonomic nervous system stress-related functions (parasympathetic, sympathetic, and polyvagal), may be measured by the heart rate variability (HRV; Arakaki et al., 2023). HRV is a measure of the variability of heartbeat-to-heartbeat intervals. It represents the capacity for self-regulation, as measured by the dynamic interface of the parasympathetic and sympathetic nervous systems. When controlled for age, higher HRV and coherent, meaning rhythmic repeated HRV patterns, are a measure of wellbeing (Arakaki et al., 2023). In contrast, age-controlled low-HRV numbers and disorganized HRV presentation are characteristic of anxiety, traumatic presentations, and medical illness (McCraty and Shaffer, 2015). Optimal HRV function is not a measure of relaxation; instead, it is a measure of the autonomic nervous system's dynamic, flexible, and appropriate function that contributes to alertness, decision-making, and expective function. Creativity has also been associated with a ruminate of associative and cognitive fluid states, leading to creative coherent autobiographical solutions (Jung et al., 2013; Marron et al., 2020; Spreng and Grady, 2010). HRV training has also been linked to a promising treatment for PTSD (Burback et al., 2024). From an ATR-N perspective, it is likely that using sensory-enriched materials supports HRV functionality and coherence (Czamanski-Cohen et al., 2020; Haiblum-Itskovitch et al., 2018). HRV functionality and coherence have also been associated with differences in capacity for resiliency and empathy, with higher HRV variability associated with increased capacity for compassion (Lischke et al., 2018). From an art therapy media perspective, Hass-Cohen et al. (2022c) have also demonstrated that using fluid materials, such as water, paint, and clay, supports a coherent emergence of compassion and empathy, two ATR-N therapeutic principles (Hass-Cohen, 2007). It is not clear whether and how self-selected familiar vs. non-familiar materials or structured or fluid materials impact coherency (Haiblum-Itskovitch et al., 2018; Hass-Cohen et al., 2022b; Lynar et al., 2017).

## ATR-N MR intervention protocol: conditions and sequencing applications

As stated earlier, several versions of the ATR-N MR protocols were tested empirically, but they all followed the same MR conditions. A small and tangible reminder and the perception of the problem (or of the self) are accessed before and after depicting resources that address the problem (new information intended to trigger a prediction error and a corrective emotional experience), followed by an opportunity to further modify the presentation of the problem or the self. What is meant by a small reminder is that it does not include all the details of what has happened. Asking for such an incomplete, that is, interrupted, reminder can function in and of itself like a predictive error (Bavassi et al., 2019). In summary, a small reminder of the problem is therapeutically established, and then the new information (Hupbach et al., 2007), facilitated by ATR-N CREATE interventions, may modify, supplement, or erase the original memory response (Hass-Cohen, 2024; Table 1).

**Table 1:** Memory reconsolidation CREATE ATR-N conditions and sequencing (see Table 2 for corresponding prompts).

**Therapeutic ATR-N-MR sequence**				
**Sequencing**		**Tasks**	**Conditions**	**ATR-N**
1.	Preliminary	Establish therapeutic relationship and collect presenting problem data.	Avoid traumatization.	Relational responding
		Collect autobiographical art-based timeline using structured media, such as pencils, markers, etc.	No treatment offered in preliminary sessions and information, or history is not explored	Adaptive responding & Empathizing compassion
2.	Beginning Treatment: Recall	Identify and prioritize main problem and related sub-problems	Identify low to mid-level arousal to current problems and situations	Creative embodiment in action
		Establish the current distressing memory or sub-memory reminder by drawing that can be used for safe and non-kindling recall as demonstrated later in the case illustration	Identify vague or vivid memories	Expressive communicating
		Using structured media, such as pencils, markers, cutouts, etc.	Emphasize safety Avoid re-traumatization	
3.	Mid-Treatment: Update	Generate novel information by identifying, activating and exposing prediction errors by pairing and disconfirming stuck belief, cognitive and emotional systems	Engage in exploration of unique outcomes and experiences or successful positive schemas that have been set to the side	Transformative integration
		Prompt for the representation of and identification of prediction errors such as the depiction of optimism resources, resiliency and success	Update negative stuck schemas and ongoing repetitive fears, stress and traumatic reactions and experiences with resilient responses	
		Use multiple and if possible unfamiliar to the person media		
4.	Retention	Protect window of change of four to six hours	Control for retroactive interference	Adaptive responding Empathizing and compassion
		Keep art-based representations	evidence of MR in a safe and protected space	Document creative expression and encourage a preference for predication errors
5.	Generalization	Repeat: Continue to reinforce and find additional prediction errors and disconfirming information	Repeat MR conditions as above	CREATE
		Use Expressive art to document success and changes. Multimedia of persons choice	Collect disconfirming information, and prediction errors	

CREATE: Creative embodiment in action, 2) Relational responding, 3) Expressive communicating, 4) Adaptive responding, 5) Transformative integration, and 6) Empathizing and compassion (CREATE). ATR-N column: Main principles listed for related art therapy interventions (Hass-Cohen and Clyde Findlay, 2015).

The sample case protocols that follow are based on the researched ATR-N protocols and guidelines for MR conditions. Consent for publication and institutional review board approval were obtained.

## Protocols case example

Sue, a woman in her late 50s with a history of PTSD, completed two protocols. The first was completed in 1.5 h over a period of 2 days and the second in a 1-day 2-h meeting. Sue requested to participate in the second protocol after realizing the benefits of the first meeting and the intrusion of a traumatic memory.

The first three prompts were to represent *the problem, the internal and external resources that help/ed with the problem*, and then to *represent the problem as you see it now*. A digital image of the resources was taken after it was completed to enhance its influence. The fourth prompt was to *check, change, and keep what you need/want of the problem representation in the third prompt*. Sometimes, a digital image can be taken of the third prompt representation if the person wants to alter it. The diverse materials included white and colorful construction paper, oil pastels, and markers, as well as other playful crafty items. After drawing, a title and a narrative were asked for. The first drawing was not discussed and was placed, facedown, to the side. Additional media from the crafts selection were offered for the second prompt. To establish context (Hupbach et al., 2008) and new meanings, a discussion was initiated after drawing the resources.

Questions focused on identifying the order in which the resources were drawn, examining which ones were external or internal resources, reviewing the order in which they were drawn, and reinforcing which resource seemed at the time and now the most significant. There are no right or wrong responses to which resource is external or internal, nor should this identification be evaluated or interpreted. Rather, the purpose is to thicken the discussion to modify the memory. It is with this same purpose that frequency, intensity, duration, and onset, as well as each resource's meaning, are investigated. Once this exploration is completed, the next drawing prompt is provided while the resources drawing is kept visible. The purpose is to run interference with the distressing memory. After the full protocol is drawn, all the presentations (Prompts 1–4) are looked at and discussed again. A comparison of the different problem representations or any repetitive prompt is invited for discussion (Table 2).

**Table 2:** Protocol.

**1. Represent the problem**
Purpose: MR short reminder
**Media:** A thick stack of 11″ × 14″ construction papers.
1. Name the problem that you would like to work with today.
a. Discourage descriptions ask for a name, title.
2. Choose one 1/2 page of colorful construction paper for a background (out of a stack of 1/2 page colorful construction paper).
a. Full pages (8.5″ × 14″) are cut in half as the directive is provided. Splitting the page in half while providing the directive is intended to communicate that that there is not much space allotted to the depiction of the problem.
3. Represent the event/problem by making cutouts from the 1/2 size construction paper (8.5″ × 5.5″).
a. While making the art a verbal reminder is given twice that it would be possible to “do this” in a short amount of time and that there was no need to represent all the details.
b. After ten minutes, s request to stop making the art if given.
4. Make a folder in which you place what was created. Take the folder with the image inside and place it in a safe place in the room.
a. A choice of 11″ × 14″ multi-color construction paper and a stapler are provided which are used to create the folder.
b. As the problem image is placed in the folder the stack of colorful half pages of construction paper and excess cuttings are removed from the art making table. Instead, the media for directive two is placed on the table.
5. No discussion of the art is initiated in order to minimize reinforcement of the memory of the problem.
**2. Represent your internal and external resources that helped with the problem**
**Purpose:** Updating problem, pairing it with new or recalled resilient past or disconfirming information.
**Media:** A thick stack of 11″ × 14″ construction papers and tissue papers
1. Represent your internal and external resources that helped with the problem using paper cut outs.
a. A thick stack of 11″ × 14″ construction papers and tissue papers are already on the table.
b. Inquiry
i. Share what each cut-out means (open ended).
ii. Then, name all of the resources, and label them as internal or external (some may be both internal and external. It is up to you to decide what is internal or external for you)
iii. Which one of these resources is the most important to you? (out of all the internal and external resources), and please explain why this is the most important to you?
iv. Please identify which one is the second most important resource for you? Please explain why this is the second most important for you?
v. Please identify which one is the third most important resource for you? Please explain why this is the third most important for you?
vi. What one did you make first, second, and so on? Which resource is most important”?
2. 10-min break
**3. Represent the problem as you see it now**
**Purpose:** Checking for changes in the updated problem representation.
**Media:** A thick stack of 11″ × 14″ construction papers, tissue papers, and oil pastels
1. After coming back from break take a digital image of the directive 2 art.
2. Leave directive 2 art on table.
3. Now: Represent the problem as you see it now.
**4: Check, change, keep what you need/want from the revised event/problem representation 3 or create a new image**.
**Purpose:** Re-checking the updated problem representation
**Media:** A thick stack of 11″ × 14″ construction papers and tissue papers, oil pastels, and markers offered.
1. Directive 3 art is on the art table.
2. The art is changed or created anew as needed

### Case study protocol 1: coming down the mountain

**Figure 4:**
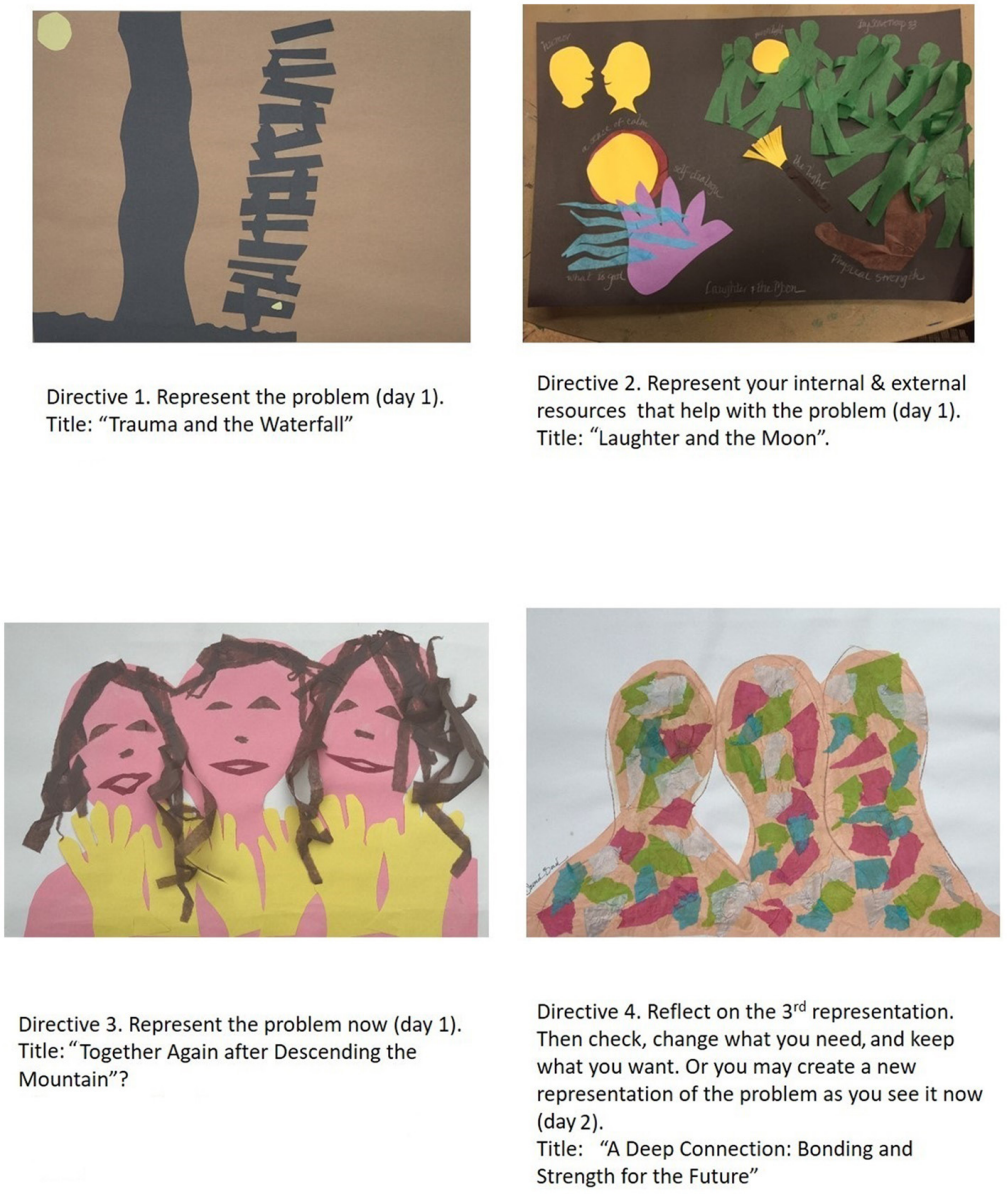
Protocol 1: coming down the mountain.

At the first meeting, Sue was asked to “*represent the problem*.” She chose a half-page of black paper for her background out of a stack of colorful, half-page-sized construction paper. As she was drawing, she was reminded that “it would be possible to complete the image in a short amount of time and that there was no need to represent all the details.” Sue created “Trauma and the Waterfall” (Prompt 1), which represented an event that happened 17 years ago. It depicted her and her children's descent from a steep mountain. Coming down the slope, the family members rushed to reach the end of the path before dark. They walked along steep, wet stairs in total darkness next to a raging river without a flashlight. After 10 min, Sue titled her art and was asked to place it in her construction paper folder, which was set aside without further discussion (Figure 4).

Sue then used paper cutouts to “*represent the internal and external resources that helped her with the problem*.” “Laughter and the Moon” represents her internal resources: finding humor in the situation, positive self-dialogue, and crisis skills. Calmness and her ability to soothe her daughter were her most important resources. Sue's external resources were represented by green tissue paper figures symbolizing the bright moon, the flashlight, her physical strength, and the Boy Scout troop, which had escorted them safely down the mountain back to camp.

After a 10-min break, Sue took a digital picture of the resource representation artwork (Prompt 2). The art then remained on the worktable, and she created “Together Again after Descending the Mountain.” This representation was in response to the third prompt, “*represent the problem as you see it now*.” Her image included yellow cutouts of hands representing the yellow light of the flashlights and how the family members held hands as they descended the wet stairs. She mentioned that she was not happy with the image, as the faces had a surreal, clown-like look to them and the hands looked more like yellow rubber cleaning gloves. Sue commented that she found herself in a hurry to finalize the piece and to put it away.

On the second day, Sue was asked to look at her image “Together Again after Descending the Mountain” and “*check, change, or keep what she needed and wanted from the image or create a new one*.” She reported that she was surprised by the unanticipated flood of emotions and physical fear associated with the image that she had created. She pondered why she had used red, yellow, and black—colors she strongly disliked—for the three family figures. She recollected that she associated these colors with a physical assault she had suffered 13 years ago, 4 years after descending the mountain. She reported realizing that the same internal and external resources had helped her survive the mountain as well as the assault. She had remained calm during the assault, her physical strength helping her fight for life. She said that the green figures transformed into a memory of eight police officers (from the other trauma) who rescued her. Sue then created a final image, “A Deep Connection: Bonding and Strength for the Future,” representing a deeper, post-trauma connection to her now adult children. Reminiscent of “Together Again after Descending the Mountain,” “A Deep Connection: Bonding and Strength for the Future” is adorned with softer colors, which she said she found soothing and strengthening, evoking lighter feelings of calmness and peace.

Two weeks later, Sue reflected that as she now recalled the memory of the mountain event, she did not experience any emotive or physical arousal: “It is as if my responses have smoothed out, taking on a softer form.” She reported her current level of arousal of 4, as compared to an 8 or 9 during the first recall. Sue also shared that her intention to remember and process a small event that occurred with her children also brought up a different larger trauma, an assault, which she said emerged in “[her] color choices.” Sue added that it was the disturbing colors, and strangely enough, her recall of the support from her children was unconsciously brought up the implicit memory of the assault. Their current meaningful relationship continues to support her now that she has recalled the severe attack. She asked to repeat the protocol to focus on the memory of the assault that intruded into the memory of coming down the mountain.

### Protocol 2: the assault

**Figure 5:**
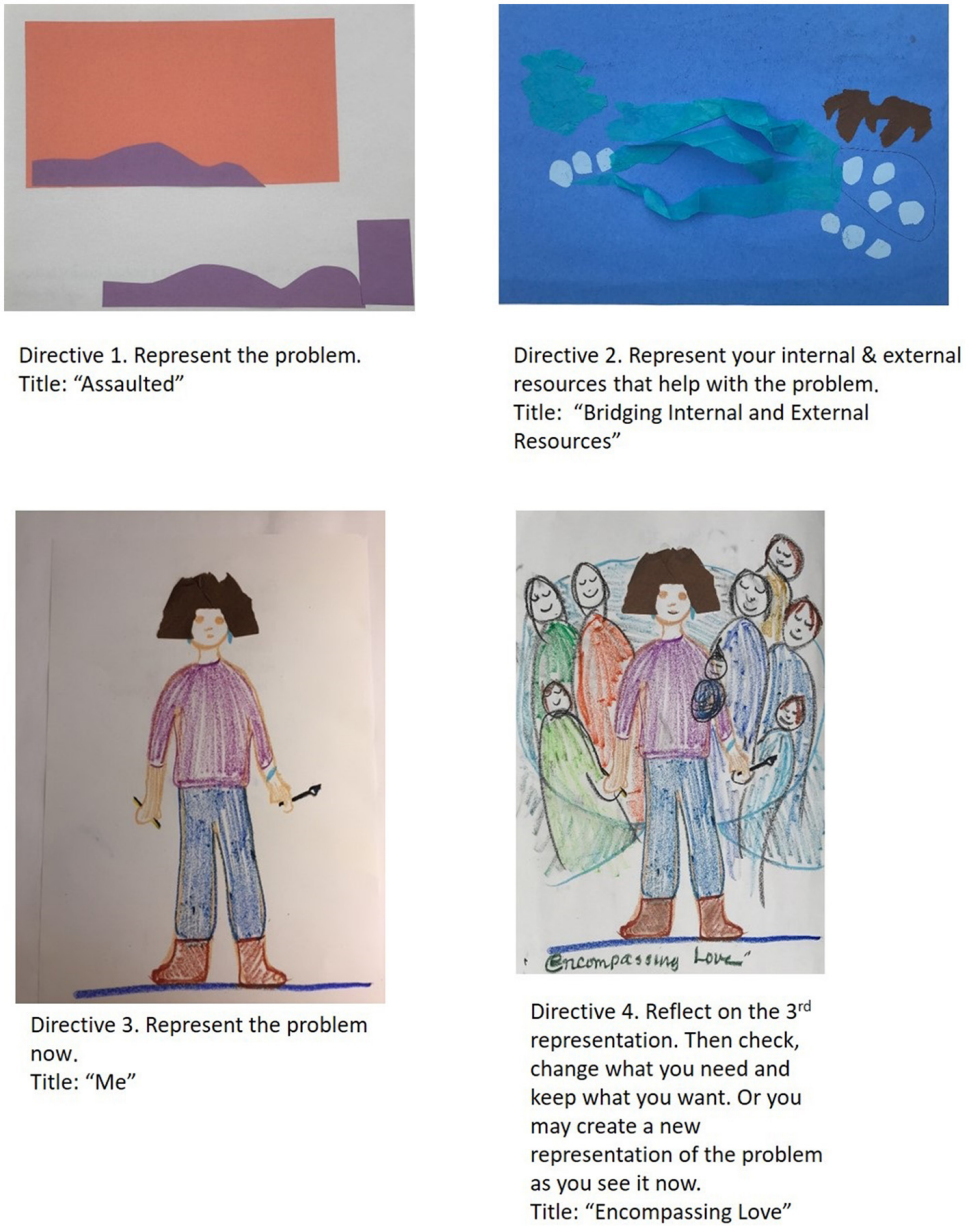
The assault (protocol 2).

In “Assaulted,” Sue first used cutouts to “*represent the problem*,” herself lying on the ground on the bottom part of the page. She reported being semi-unconscious during the attack. The top portion of the first representation shows her again. This time, the purple figure is enclosed in a small orange closet where she had found refuge. Despite the physical assault, pain, and shock, she spontaneously said that during the attack, her “stubborn” traits allowed her to think and act proactively and protectively (Figure 5).

For the resources prompt materials, such as tissue paper were introduced as novel materials for her protocol. In the next drawing, “Bridging Internal and External Resources,” a fist-like blue tissue paper shape on the top left of the drawing and three lighter dots represented Sue's fighting spirit and her mother's and children's support (Prompt 2). During the attack, she called on self-defense moves and mental strength. Smiling, she said that as the police were wheeling her away, she told them that they should be careful about secondary trauma. She had just learned about that. Sue reflected that her care-taking interests were part of her family-of-origin role. She added that after the attack, she retreated to her home and spouse. On the right side of the page, two brown shapes represented her two large and protective dogs, whereas the eight light blue dots were representative of supportive community members. As the discussion of the art revealed a spatial distance between Sue's external and internal resources, she joined the two with a blue turquoise bridge. She reflectively noted that the color turquoise was part of her heritage, which she integrated into the blue background of the art and her turquoise jewelry.

After taking a digital image of her representation of internal and external resources (Prompt 2), Sue was asked to “*represent the problem again*” (“Me,” Prompt 3) with oil pastels, which were offered as a novel material for the protocol. Sue said that oil pastel crayons were a more familiar medium, and she seemed surprised and happy to have them. In “Me,” Sue holds a brush, symbolizing a live-model drawing class that she took after the assault, which desensitized her fear of males. In her right hand, a pen represents graduate school, which she continued to attend, despite her injuries. A brown tissue paper cutout represents her current haircut, which adorns the figure from the past.

The fourth and last prompt was “*check, change, keep what you need/want of the second event/problem representation*.” Sue added support to “Me,” including family figures and a blue line, which was transformed into a quilt to surround her. Sue mentioned that the circular shape of “Encompassing Love” was suggestive of her mandala art. Then noticing that her haircut tissue cutout had a tear, she lovingly fixed it.

As Sue talked, she shared that she and her husband separated about 6 months after the assault and that, since the assault, she had avoided dating. She said that it was possible that this avoidance was unknowingly supporting the assault memory and that now that she has “faced it” she is thinking that the hidden triggering symptoms will be gone. Two weeks later, Sue reflected on her emotional reaction to the recall of the memory of the assault. She said that realizing that her avoidance of dating was keeping the memory of the assault alive was a profound insight, and she reported no arousal associated with the assault.

It is curious that the presentation of the problem has shifted to a representation of herself. This suggested that she updated the memory of the assault with an integrated self-image that included her art and writing interests, and her social supports. The figure is now standing in a strong face forward position. The problem was no longer depicted as something that had happened to her, striking her down to the floor in a victim pose as in “Assaulted;” rather, it now depicted her survivor stance. This survivor stance was also represented by a change in the paper orientation, which shifted from landscape to portrait, as well as in her body position, which shifted from supine to upright.

## Discussion

### Sequencing

The ATR-N-MR sequenced protocol can be completed in one or a few meetings. For research purposes, it is optimal to complete the protocol in a single meeting that consists of 90–120 min. For both clinical and research applications, a 10-min break can be provided as necessary. If administering the protocol in two meetings, ensure the first meeting (after completing the first two prompts) ends positively to avoid traumatization. One possibility would be to add another prompt after the second one, requesting the person to represent something that has helped them with the problem. Then the second meeting can start with the prompt for resource representation. To avoid kindled traumatization, the sole and uninterrupted application of the first prompt (the reminder) without pairing with some new meaning is strongly not advised (Lane et al., 2015). The person's response and pace should be taken into consideration. If the person is very overwhelmed by the first and second prompts, clinicians should use their clinical judgment to remediate these issues. These decisions are based on therapeutic and sequencing considerations. The sequenced revisiting of the representation throughout the protocol allows for MR modification of the problem to occur at least three times, in each representation of the problem (Hass-Cohen, 2016; Hass-Cohen and Clyde Findlay, 2019b; Hass-Cohen, 2024). One reason for these repeated attempts at modifying the memory is that negative-based memories, which are preferentially encoded and resistant to change (Schwabe et al., 2014). While the first prompt invites a brief recall of the problem, the following prompts support progressive exploration and establishment of new meaning. Not only the prompts but also the media provide an avenue for memory modification. While, for the first prompt, a small-sized paper is available, full-sized paper and added novel media choices are provided for the following prompts. Prompts, processes, and media choices are designed to ensure a safe and therapeutic experience (Hass-Cohen and Clyde Findlay, 2015). The protocol's specific prompts might be perceived as unexpected, potentially causing mild stress while stimulating curiosity and imagination.

Additionally, integrating experiences of safety, resource exploration, curiosity, and optimism with the problem recall and its expression is uniquely designed to trigger prediction errors (Blackwell et al., 2013; Hass-Cohen et al., 2022c,b).

### The reminder

The recall of the distressing memory or the issue is activated by a reminder, which is embedded in the first prompt: “represent the problem.” The use of this language is intentional as what had happened is not precisely identified other than “the problem,” or “what has been happening,” or “what is bothersome.” The purpose of this strategy is to curtail arousal and rekindling (Barreiro et al., 2013), as well as allowing for an expression of the here-and-now impacts of the past event/issues and for implicit unexpressed and unnamed issues to emerge and be addressed during the ATR-N MR processing (Hass-Cohen, 2024).

Arts therapies strategies may support these purposes by using materials and arts-based structured interventions to carefully and safely create a vivid representation of a distressing problem (Gerge and Pedersen, 2017), which may assist in the recall of vague long-term memories and make them available for MR modification (Hass-Cohen and Clyde Findlay, 2019b). To manage hyperarousal, which can be triggered by vividness or detail, the size of the paper can be controlled by several strategies. Colorful construction paper is used for the protocol, and the person is invited to select the color of the paper that they would like to use before they hear the first prompts. Frequently, people will usually choose colors that they like or a color not associated with the “problem” or a distressing memory as the first prompt has not yet been requested. This may create a prediction error or a mismatched representation of the experience as the subsequent image of the distress may be dissonant with the colorful and possibly positive background. For the first prompt, the size of the construction paper to be used for the subsequent prompts is cut into two, and a suggestion is made after the prompt is provided: “There is not much space for this drawing, and you can finish it quickly.” The purpose is to discourage prolonged and detailed recall. Limiting the time for the first prompt drawing is recommended. While the person is asked to title the drawing, a detailed narrative is not encouraged, and the representation is turned over and put to the side. For the first prompt, arousing forms and colors can be contained by offering black and white colored markers and suggesting an abstract presentation by encouraging the use of paper cuts. The ease with which a cutout is created may decrease stress, negative emotions, and judgment and may provide a sense of safety and support through familiar artmaking procedural skills involved with cutting paper (Hass-Cohen and Clyde Findlay, 2015). Conversely, because procedural memories (such as cutting and pasting) are automatic responses that do not require any attention or provide a distraction, they may easily allow for memories to arise (Hass-Cohen and Clyde Findlay, 2015). Other distancing, regulatory, and mitigating strategies include asking the person to visualize projecting a black-and-white image of what they want to present, either as if on a screen or from a bird's-eye view (Gray and Liotta, 2012). The latter approach has been reported to reduce the traumatic symptoms of PTSD. A depersonalized bird's-eye vantage point is frequently used by trauma clients. Sue used this viewpoint spontaneously without any instruction for the first prompt. If the person wants to narrate the first prompt, ask them to describe and label it instead. Assure them they will be able to narrate it fully by the end of the meeting. Additional verbal therapeutic interventions should provide reassurance to the individual that they are supported and secure in the present moment. These cognitive and emotional strategies are designed to counteract the reactivation of neuropathways that sustain traumatic memories (Hass-Cohen, 2016).

## Prediction errors: pairing old and new evidence

The invitation to represent the “internal and external resources that help or helped with the problem,” aims to modify the memory of the problem by creating prediction errors.

Prediction errors can be triggered by pairing the memory of the problem with disconfirming facts such as past or current resources (Ji et al., 2022). Pairing a traumatic memory with the pleasure of creative artmaking is fundamental to arts therapies. The juxtaposition of negative and positive experiences scaffolds MR prediction errors, reducing the strength of trauma reminders as well as arousal and cognitive distortions. Following the art making, discussing the art product consolidates the novel resource-focused memory. Expressed verbal listing of the implicit art-based representation of the resources facilitates explicit awareness and growth of coping abilities and resilient responses. This process helps disconfirm the problematic memory and affirm the updated memory. The visual-spatial organization of the resources on the page further enhances prediction errors and perhaps disrupts hippocampal patterns. An example is Sue's organization of her resources, which included light in the first protocol that served as a dynamic spatial focus and a bridge to her family in the second protocol, thereby connecting the two parts of her representation. It is possible that Sue's recall of social interactions also supported the scaffolding of prediction errors (Heffner et al., 2021; van Leeuwen et al., 2022). She recalled an embodied sense of community support, which likely reconsolidated as an experience that is counter to the social isolation frequently reported by trauma survivors. The sensory and tangible reminder of her children's family and neighbors' unconditional love, which did not require sharing of the details of what had happened, likely helped resolve her internal conflicts and ignite some self-compassion and bonding hormones (Blackwell et al., 2013; Patin et al., 2018).

## Reconsolidation: updated memories

Once the resource representation is completed, it is placed in full view and remains visible to the person during the response to the next two prompts. The aim is for resilient resource memories to intrude on the invitation to “represent the problem now.” These kinds of intrusions, which function to update the memory, have been associated with the memory reconsolidation process (Capelo et al., 2019; Hupbach, 2011). By emphasizing the present tense (“now”), the prompt encourages a reexamination of the problem within the context of available resources (Bridgham and Hass-Cohen, 2008; Hass-Cohen, 2008). This principle is applied across all possible protocol variations (Hass-Cohen et al., 2018, 2022c), for instance, when a review of the self in the context of trauma and then in the context of resources is requested in Prompts 3 and 4 (Hass-Cohen and Clyde Findlay, 2019a). Often, as in Sue's case, reexamining the problem emerges as a representation of the self within the context of the resource. Both revisiting of the problem (or the self as in the other protocol variation) may also be conceptualized as a representation of internal working memory security (Hass-Cohen and Clyde-Findlay, 2009; Hass-Cohen and Clyde Findlay, 2019a; Leyh et al., 2016). Sometimes, significant attachment figures, which may assist in resilient recovery (Selcuk et al., 2012) emerge (Hass-Cohen and Clyde Findlay, 2019b). A further comparison of the first and second representations of the problem in the last, fourth prompt is suggested: Check, change what you need, and keep what you want (from Prompt 3), or create a new representation of the problem as you see it now. The last prompt overtly calls for a cognitive examination of potential changes and modifications. It provides an additional opportunity for engendering and establishing prediction errors by including memories of resilient and resourceful coping, as well as by pairing negative and positive reactions. To her last drawing, Sue added contextually anchoring details including a background. She incorporated her social support and recalled her artistic capacities as well. She also carefully repaired her self-representation. This attention to detail and artistic perspective may be associated with posttraumatic growth and resiliency, a return to baseline function, and reengagement with complex thinking. The latter is often limited by trauma-induced black-and-white thinking (Brewin, 2018).

### Reaffirmation

The last prompt, “check, change what you want, and keep what you need or make a new image,” addresses any residual negative effects or cognitions that need attention and provides an opportunity for supportive changes. The altered artwork is then discussed to augment and solidify these changes. The altered artwork serves to continue and document this potential. The purpose is to continue pairing aspects of the problem with personal resources, the capacity for change, and the artmaking experience. Using the word *need* is also intended to prompt self-care and possibly self-compassion (Hass-Cohen and Clyde Findlay, 2016). For example, when checking her image in the second protocol, Sue tenderly mended the tear in her tissue paper “hair.” Rechecking the second representation of the problem provided an opportunity for a tangible recording of her perceived changes to the title and narrative, which is encouraged for each of the three prompts (2, 3, and 4). Discussing each of the prompts and reviewing the complete protocol helps to reaffirm the understanding of the problem within the context of personal and social resources and creativity. The identification and discussion emphasize personal strengths, traits, and resources whether occasional or enduring and typical. To further enhance therapeutic change and resiliency, factors such as optimism, hope, courage, or creativity are explored (Fredrickson, 2013).

## Discussion

MR neuroscience research is continuing to shed light on the theory and practice of arts therapies (Kindt and Elsey, 2023). Specifically, it has aided in revisiting and establishing MR as an ATR-N change factor. Prescribed ATR-N MR conditions have underscored the relevance of accurately and safely managing sequencing, problem recall, and prediction error activation. While these conditions have been discussed in the context of the ATR-N protocol, they are relevant to diverse art therapies practices. By virtue of artmaking, these facilitate the updating of recalled memories with novel and mismatched experiences. Overall, when compared with verbal discussion, expressive and creative activity has been identified as a critical agent of change and resiliency (Hass-Cohen et al., 2018, 2022a). The advantage of the ATR-N MR protocol is the continuous reiteration and strengthening of updated explicit and implicit memories. After implicit and explicit trauma and pain memory recall, the imagery and tangible arts therapies protocols may soften and change the harsh recall with novelty (Hass-Cohen, 2018). The protocol sequencing is designed so that visual, sensory, affective, and executive experiences trigger a retroactive MR interference and update. Stimulating the creative response further focuses interest on the MR processes. The tangible art products that can be reviewed during and after the completion of the work continue to support autobiographical resilience and identity, which serve to mitigate PTSD symptoms (Hass-Cohen et al., 2022c). However, due to proactive interferences of implicit and explicit negative biases and to discover what is really bothersome and distressing, the protocol may need to be repeated.

Nevertheless, it is possible that distressing memories may be interrupted by a onetime visual-spatial session (Fort et al., 2023). These ideas align with the ATR-N principles, which suggest that utilizing imagination and creativity is transformational (Hass-Cohen and Clyde Findlay, 2015). Continuing to research the protocol with a diverse population and a larger sample, which may require refining the protocol and examining MR as an agent of change, is important (Dunbar and Taylor, 2017; Kindt and Elsey, 2023). Specifically, sensitivity to the level of trauma and the characteristics of memory recall, as well as cultural norms and taboos regarding self-disclosure of traumatic and distressing events, may require slower sequencing and less direct prompts. Finally, furthering brain and clinical evidence–based art therapy research is necessary to continue developing and substantiating the proposal that MR is a change factor for ATR-N approaches.

## Data Availability

The raw data supporting the conclusions of this article will be made available by the authors, without undue reservation.
